# Supplementary material for: Demographic inference through approximate-Bayesian-computation skyline plots
Source: PeerJ. 2017 Jul 18;5:e3530. doi: 10.7717/peerj.3530 (PMC5518730; doi:10.7717/peerj.3530)

Supporting Information for: Demographic inference through approximate-Bayesian-computation skyline plots

Miguel Navascués[1,2], Raphaël Leblois[1,2], and Concetta Burgarella[3]

[1]INRA, UMR CBGP, F-34988 Montferrier-sur-Lez, France.
miguel.navascues@inra.fr
[2]Institut de Biologie Computationnelle, F-34090 Montpellier, France
[3]IRD, UMR DIADE, F-34394 Montpellier, France

S1 Supplementary Methods

S1.1 Approximating $\theta(t)$ from parameters $\theta_0, \theta_1, ..., \theta_n$

In order to work with a set of common demographic parameters that will represent the demographic trajectory $\theta(t)$, the parameters of the constant piecewise demography $(\theta_0, \theta_1, ..., \theta_n)$ are transformed to parameters $\{\theta(t_1), \theta(t_2), ..., \theta(t_m)\}$ as described in the main text. A graphical representation of this transformation is provided in Supplementary Figure S1, which shows the equivalence between the original and the derived parameters for three example simulations.

Figure S1. Approximation of $\theta(t)$ from original parameters. Three simulations of demographic histories are represented, with one, none and two population size changes, respectively.

	Original parameters				
sim	θ_0	τ_1	θ_1	τ_2	θ_2
1	4	1.4	40		
2	1.5				
3	0.04	0.4	11	2.3	178

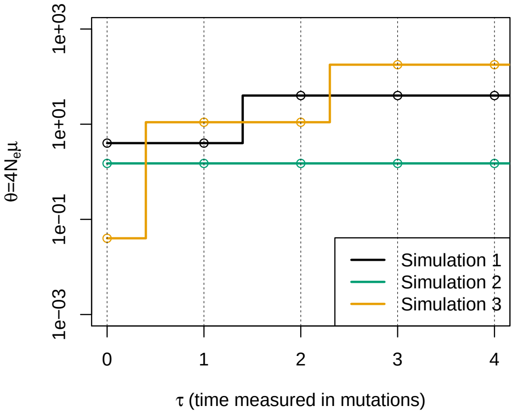

	Approximation of $\theta(t)$				
sim	$\theta(t=0)$	$\theta(t=1)$	$\theta(t=2)$	$\theta(t=3)$	$\theta(t=4)$
1	4	4	40	40	40
2	1.5	1.5	1.5	1.5	1.5
3	0.04	11	11	178	178

S1.2 Alternative ABC skyline plot

In addition to the procedure described in the main text, other ways to obtain skyline plots in approximate Bayesian Computation (ABC) can be devised, particularly by changing the number and type of models and the prior probabilities of parameters. For comparison, we present one of these alternatives. In this ap-

proach we consider a single piecewise model with a single demographic change. The parameters of the model are θ_0, θ_1 and τ_1, following the nomenclature presented in the main text. Demographic inference is performed on the derived parameters $\theta(t_j)$ in the same way as described in the main text. Evidence for population size change will be considered by evaluating the ratio θ_0/θ_1 in a similar way as in MIGRAINE. This alternative approach was evaluated on the three example scenarios (contraction with $\theta_0 = 0.4$, $\theta_1 = 40$, $\tau = 0.1$, expansion with $\theta_0 = 40$, $\theta_1 = 0.4$, $\tau = 0.1$ and constant size with $\theta = 40$; mutational model with $P_{GSM} = 0.22$). Simulated data sets were generated for 50 individuals genotyped at 30 microsatellite loci.

S1.3 Comparison with alternative methods

We present a comparison of results between methods implemented in four programmes: DIYABCskylineplot (Navascués, 2017), MIGRAINE (Leblois et al., 2014), VarEff (Nikolic and Chevalet, 2014) and BEAST (Drummond et al., 2012), which can make inferences of demographic change from microsatellites following a generalized stepwise mutation model (GSM). This is not intended to be a thorough cross comparison, but to provide some intuition about how different their results can be, their limitations and, thus, the potential interest of their simultaneous use. We knowingly exclude MSVAR (Beaumont, 1999; Storz and Beaumont, 2002) from this comparison because it only implements a strict stepwise mutation model (SMM). We also excluded methods based on summary statistics (Cornuet and Luikart, 1996; Kimmel et al., 1998; Garza and Williamson, 2001) as we considered them to be superseded by ABC inference. However, Girod et al. (2011) evaluate those methods on equivalent simulated scenarios. A more thorough evaluation of the methods implemented in MIGRAINE and VarEff under other scenarios can be found in the original publications (Leblois et al., 2014; Nikolic and Chevalet, 2014). To our knowledge, there is no work addressing the performance of the extended Bayesian skyline plot method included in BEAST on microsatellite data.

All four methods have been used to analyse seven data sets, three simulations (PODs, pseudo-observed data set) and four real data sets presented in the main text (leatherback turtle, whale shark, black-and-white colobus and red colobus). The three PODs that we evaluated correspond to the example scenarios used through this work (contraction with $\theta_0 = 0.4$, $\theta_1 = 40$, $\tau = 0.1$, expansion with $\theta_0 = 40$, $\theta_1 = 0.4$, $\tau = 0.1$ and constant size with $\theta = 40$; with a generalised stepwise mutational model (GSM) with $P_{GSM} = 0.22$). Simulated data sets were generated for 50 individuals genotyped at 10 microsatellite loci. The reduction of the number of loci respect to the simulations presented in the main text was decided to reduce the computing time of the analysis with BEAST.

Models and assumption of the methods implemented in DIYABCskylineplot and MIGRAINE are described in the main text. The analyses of the four real data case studies correspond to those presented in the main text. For the analysis of the three PODs, DIYABCskylineplot was run with the same priors

described in the main text; and MIGRAINE was run using 2000 trees, 400 points at each iteration and a total of 12 iterations.

We used BEAST v1.8.0 (Drummond et al., 2012) to perform an extended Bayesian skyline plot analysis (Heled and Drummond, 2008). The general approach is, in several ways, similar to the one used in our ABC analysis but implemented in a MCMC coalescent sampler. The main difference is in the demographic model, which is a linear piecewise model (instead of constant piecewise model). That is, the model is defined by the scaled effective population size values θ_i at n times points ($i \in [0, n]$). Effective population size changes linearly from θ_i to θ_{i+1} between consecutive time points i and $i + 1$. The number of time points defining the model is explored using a Poisson distribution, which allows discriminating between stable and changing demographies as described in the main text. Regarding the microsatellite mutational model, BEASTS v1.8.0 allows for complex features, such as mutational bias between insertion and deletions of repeat units, allele size dependency of mutation rate and presence of multi-step mutations (Wu and Drummond, 2011). We have limited our analysis to the model equivalent to the GSM (called two-phase model with a single parameter in BEAST). For each data set, we run three independent chains of 10^9 steps, sampling parameters every 10^5 steps and discarding the first 10^8 steps as a burn-in, except for the red colobus for which three additional chains were run to achieve effective sample sizes larger than 100. Due to the computational burden of the analysis, we opted to reduce the sample size to 50 random individuals for the whale shark and leatherback turtle (original sample sizes were 478 and 215 individuals respectively) and to fix the GSM parameter to $P_{GSM} = 0.55$ for the whale shark and $P_{GSM} = 0.56$ for the leatherback turtle. These values were based on the estimates obtained with DIYABCskylineplot and MIGRAINE (see Table S3 and Figure S16). For the POD of a contracting population, due to problems of convergence and computing time, the P_{GSM} was also fixed to the true value 0.22. For the rest of the analysis (colobus species and PODs from expanding and constant population) we used a uniform prior between zero and one for the P_{GSM} parameter. All other options and priors were left to its default values.

The forth method is the composite-likelihood approach implemented in the R package VarEff (Nikolic and Chevalet, 2012). In this method, the data are reduced to the distribution of pairwise differences (difference in number of motifs) among gene copies for the sample. The probability that two gene copies differ by x number of motifs given a constant piecewise demographic model with parameters $\{(\theta_i, \tau_i); i \in [0, n]\}$ and a GSM with parameter P_{GSM} can by calculated through numerical integration (Chevalet and Nikolic, 2010). Nikolic and Chevalet (2012) exploit this result to estimate the parameters of the demographic model with an MCMC algorithm. However, the mutational parameter P_{GSM} is considered to be known and it is not estimated but fixed *a priori*. We run this analysis considering a model with three population size changes, uniform prior for the time $\tau = t\mu$ between 0 and 4 (to explore the same time interval as with the ABC method), mutational parameter fixed to $P_{GSM} = 0.37$ for the black-and-white colobus, $P_{GSM} = 0.215$ for the red colobus, $P_{GSM} = 0.55$ for

the whale shark, $P_{GSM} = 0.56$ for the leatherback turtle and $P_{GSM} = 0.22$ (i.e. the true value) for the three PODs. These values were based on the estimates obtained with DIYABCskylineplot and MIGRAINE (see Table S3 and Figure S16). We run a chain with 10^5 samples, separated of 100 steps and taken after a burn-in period of 10^5 steps. All other parameters were set as recommended by authors (Nikolic and Chevalet, 2014).

S2 Supplementary Results and Discussion

S2.1 Alternative ABC skyline plot

The alternative parametrization to obtain a skyline plot on the ABC framework described in Supplementary section S1.2 yielded very similar results to the approach described in the main text (Supplementary Figure S17). Credibility intervals are narrower than in Figure 1, reflecting the stronger autocorrelation of effective population size through time when only one population size change is allowed. This should not be interpreted as a better performance of the inference as it reflects the differences in the prior setting. The ability to detect population size change is similar using both sets of priors (Supplementary Figure S18).

S2.2 Comparison with alternative methods

Graphical results of the four demographic inference methods are presented in Supplementary Figure S19 for PODS and Supplementary Figure S20 for real data. Demographic parameters estimated by MIGRAINE are presented in Supplementary Table S5. Evidence for variable demography from DIYABCskylineplot and BEAST is presented in Supplementary Table S7.

Regarding the analysis of PODs, were the true values are known, DIYABC-skylineplot and MIGRAINE inferences described the three different demographies correctly co-estimating the mutational parameter P_{GSM}. Credibility and confidence intervals contained the true values of parameters and were fairly congruent between both methods. Results from the extended Bayesian skyline plot from BEAST were less satisfactory. First, for the contracting scenario, co-estimation of demography and mutational model was computationally too expensive. Fixing the mutational parameter P_{GSM} to its true value allowed finishing the computations in a reasonable time (i.e. few days) but is not a solution available for real data for which the parameter value will be unknown. For constant and expansion scenarios, demography and mutational model were co-estimated with mixed success. The expansion was well detected, though true values were consistently outside the credibility interval. In contrast, BEAST gave very strong evidence of variable demography for the constant model, with a skyline plot suggesting a demographic expansion and an estimate of P_{GSM} far from the true value. Finally, VarEff performed well for characterizing the contraction and the constant size, but no the expansion. Note that original evaluation of the VarEff method already shows some limits in the detection

of recent demographic increase, but it is able to characterize older expansions (Nikolic and Chevalet, 2014). Nevertheless, given that VarEff requires fixing the value of P_{GSM} and we used the true value, these results are not of much relevance to the application of the method to real data.

The analyses of real data with the extended Bayesian skyline plot and VarEff highlight some of the problems described above with the additional difficulty of the lack of knowledge on the true demography and mutational process. Analyses with BEAST required reducing the sample size and fixing the mutational parameter to reduce the computation for two of the datasets (whale shark and leatherback turtle). In order to fix the values of P_{GSM} for the analysis in BEAST and VarEff, we based our choice on the results from DIYABCskylineplot and MIGRAINE analysis of the same datasets. This is a practice that should be avoided in the analysis of real data and we did it as the only way to provide some comparison of results among methods. VarEff analysis did not reveal any striking demographic change in the history of those populations, suggesting a constant population size. Extended Bayesian skyline plots from BEAST were fairly flat for all four study cases and inferred systematically lower effective population sizes than the other methods. Considering the Bayes factor, evidence was either negative or weak for population size change: 1.36 for the whale shark, 1.75 for the leatherback turtle, 0.78 for the black-and-white colobus and 1.68 for the red colobus. Although the amount of data was much lower than that used by the other three methods for the whale shark and the leatherback turtle, the reduced datasets are similar in size to the PODs for which it was able to retrieve signal of demographic change.

The limited number of simulations does not allow us to conclude much about the performance of the different methods and the computation cost of BEAST precludes increasing the number of simulations. Nevertheless, this comparison is informative about the applicability of the methods to real data and their limitations. In our opinion, the use of VarEff on real data is difficult to justify, as it requires some knowledge on the mutational process that is rarely available. We want to stress that using the estimates of P_{GSM} from the same dataset by an alternative method (such as DIYABCskylineplot or MIGRAINE) is not a good statistical practice and should not be done for the analysis of real data. The extended Bayesian skyline plot implemented in BEAST can potentially co-estimate the demography and mutational process if computation resources and time are not a constraint. However, the potential biases and pitfalls of the method have not been characterized. The failure to infer the correct demography for the simulated constant size scenario is anecdotal (many replicates would be necessary for a proper evaluation), but does not give confidence in the methods. Given that alternative methods with much less computation cost and thoroughly validated exist (i.e. DIYABCskylineplot and MIGRAINE), there is no reason to invest the computational resources required for the analysis in BEAST. Note that these remarks are valid in the context of the demographic inference from microsatellite data and should not be extrapolated to other analysis that are implemented in BEAST.

References

M. A. Beaumont. Detecting population expansion and decline using microsatellites. *Genetics*, 153(4):2013–2029, 1999.

C. Chevalet and N. Nikolic. The distribution of coalescence times and distances between microsatellite alleles with changing effective population size. *Theoretical Population Biology*, 77(3):152–163, 2010. doi: 10.1016/j.tpb.2010.01.001.

J. M. Cornuet and G. Luikart. Description and power analysis of two tests for detecting recent population bottlenecks from allele frequency data. *Genetics*, 144(4):2001–2014, 1996.

A. J. Drummond, M. A. Suchard, D. Xie, and A. Rambaut. Bayesian phylogenetics with BEAUti and the BEAST 1.7. *Molecular Biology and Evolution*, 29(8):1969–1973, 2012. doi: 10.1093/molbev/mss075.

J. C. Garza and E. G. Williamson. Detection of reduction in population size using data from microsatellite loci. *Molecular Ecology*, 10(2):305–318, 2001.

C. Girod, R. Vitalis, R. Leblois, and H. Fréville. Inferring population decline and expansion from microsatellite data: A simulation-based evaluation of the msvar method. *Genetics*, 188(1):165–179, 2011.

J. Heled and A. J. Drummond. Bayesian inference of population size history from multiple loci. *BMC Evolutionary Biology*, 8(1):289, 2008.

M. Kimmel, R. Chakraborty, J. P. King, M. Bamshad, W. S. Watkins, and L. B. Jorde. Signatures of population expansion in microsatellite repeat data. *Genetics*, 148(4):1921–1930, 1998.

R. Leblois, P. Pudlo, J. Néron, F. Bertaux, C. R. Beeravolu, R. Vitalis, and F. Rousset. Maximum-likelihood inference of population size contractions from microsatellite data. *Molecular Biology and Evolution*, 31(10):2805–2823, 2014.

M. Navascués. DIYABCskylineplot v1.0.1: A suite of R scripts to perform a Approximate Bayesian Computation Skyline Plot, 2017. doi:10.5281/zenodo.267182.

N. Nikolic and C. Chevalet. *VarEff: Variation of effective population size*, 2012. URL `https://CRAN.R-project.org/package=VarEff`. R package version 1.0.

N. Nikolic and C. Chevalet. Detecting past changes of effective population size. *Evolutionary Applications*, 7(6):663–681, 2014. doi: 10.1111/eva.12170.

J. F. Storz and M. A. Beaumont. Testing for genetic evidence of population expansion and contraction: an empirical analysis of microsatellite DNA variation using a hierarchical bayesian model. *Evolution*, 56(1):154, 2002. doi: 10.1111/j.0014-3820.2002.tb00857.x.

C.-H. Wu and A. J. Drummond. Joint inference of microsatellite mutation models, population history and genealogies using transdimensional Markov chain Monte Carlo. *Genetics*, 188(1):151 –164, 2011. doi: 10.1534/genetics.110.125260.

Table S1. Estimation of mutational parameter P_{GSM}

θ_0	θ_1	τ	P_{GSM}	MAE	bias	out of CI
0.4	4	0.01	0.00	0.07	0.07	1.00
0.4	40	0.01	0.00	0.14	0.14	1.00
0.4	400	0.01	0.00	0.41	0.41	1.00
0.4	4	0.05	0.00	0.09	0.09	1.00
0.4	40	0.05	0.00	0.23	0.23	1.00
0.4	400	0.05	0.00	0.52	0.52	1.00
0.4	4	0.10	0.00	0.12	0.12	1.00
0.4	40	0.10	0.00	0.28	0.28	1.00
0.4	400	0.10	0.00	0.47	0.47	1.00
0.4	4	0.50	0.00	0.16	0.16	1.00
0.4	40	0.50	0.00	0.41	0.41	1.00
0.4	400	0.50	0.00	0.51	0.51	1.00
4	0.4	0.01	0.00	0.04	0.04	1.00
40	0.4	0.01	0.00	0.05	0.05	1.00
400	0.4	0.01	0.00	0.04	0.04	1.00
4	0.4	0.05	0.00	0.04	0.04	1.00
40	0.4	0.05	0.00	0.04	0.04	1.00
400	0.4	0.05	0.00	0.04	0.04	1.00
4	0.4	0.10	0.00	0.04	0.04	1.00
40	0.4	0.10	0.00	0.04	0.04	1.00
400	0.4	0.10	0.00	0.04	0.04	1.00
4	0.4	0.50	0.00	0.03	0.03	1.00
40	0.4	0.50	0.00	0.03	0.03	1.00
400	0.4	0.50	0.00	0.04	0.04	1.00
4			0.00	0.06	0.06	1.00
40			0.00	0.10	0.10	1.00
400			0.00	0.19	0.19	1.00
0.4	4	0.01	0.22	0.05	-0.01	0.02
0.4	40	0.01	0.22	0.07	0.05	0.01
0.4	400	0.01	0.22	0.33	0.33	0.11
0.4	4	0.05	0.22	0.06	0.02	0.04
0.4	40	0.05	0.22	0.11	0.11	0.00
0.4	400	0.05	0.22	0.38	0.38	0.00
0.4	4	0.10	0.22	0.07	0.04	0.03
0.4	40	0.10	0.22	0.14	0.13	0.01
0.4	400	0.10	0.22	0.36	0.36	0.01
0.4	4	0.50	0.22	0.10	0.09	0.10
0.4	40	0.50	0.22	0.25	0.25	0.04
0.4	400	0.50	0.22	0.36	0.36	0.02
4	0.4	0.01	0.22	0.06	-0.02	0.02
40	0.4	0.01	0.22	0.05	-0.02	0.05
400	0.4	0.01	0.22	0.05	-0.01	0.04

Continued on next page

Table S1 – continued from previous page

θ_0	θ_1	τ	P_{GSM}	MAE	bias	out of CI
4	0.4	0.05	0.22	0.05	-0.04	0.07
40	0.4	0.05	0.22	0.06	-0.04	0.07
400	0.4	0.05	0.22	0.06	-0.05	0.04
4	0.4	0.10	0.22	0.06	-0.05	0.04
40	0.4	0.10	0.22	0.05	-0.04	0.05
400	0.4	0.10	0.22	0.05	-0.04	0.12
4	0.4	0.50	0.22	0.06	-0.06	0.13
40	0.4	0.50	0.22	0.04	-0.03	0.11
400	0.4	0.50	0.22	0.02	-0.01	0.01
4			0.22	0.05	-0.01	0.04
40			0.22	0.06	-0.03	0.00
400			0.22	0.07	0.04	0.00
0.4	4	0.01	0.74	0.05	-0.04	0.03
0.4	40	0.01	0.74	0.04	-0.03	0.00
0.4	400	0.01	0.74	0.09	0.09	0.00
0.4	4	0.05	0.74	0.09	-0.09	0.06
0.4	40	0.05	0.74	0.08	-0.08	0.00
0.4	400	0.05	0.74	0.16	0.16	0.00
0.4	4	0.10	0.74	0.11	-0.10	0.03
0.4	40	0.10	0.74	0.11	-0.11	0.00
0.4	400	0.10	0.74	0.17	0.17	0.00
0.4	4	0.50	0.74	0.07	-0.03	0.04
0.4	40	0.50	0.74	0.10	-0.07	0.00
0.4	400	0.50	0.74	0.16	0.16	0.00
4	0.4	0.01	0.74	0.03	-0.01	0.04
40	0.4	0.01	0.74	0.03	-0.01	0.06
400	0.4	0.01	0.74	0.03	-0.01	0.03
4	0.4	0.05	0.74	0.03	-0.01	0.02
40	0.4	0.05	0.74	0.02	0.00	0.04
400	0.4	0.05	0.74	0.02	-0.01	0.07
4	0.4	0.10	0.74	0.02	-0.01	0.04
40	0.4	0.10	0.74	0.02	-0.01	0.03
400	0.4	0.10	0.74	0.02	-0.01	0.04
4	0.4	0.50	0.74	0.02	0.00	0.05
40	0.4	0.50	0.74	0.02	-0.01	0.11
400	0.4	0.50	0.74	0.01	-0.01	0.03
4			0.74	0.03	-0.02	0.07
40			0.74	0.03	-0.01	0.02
400			0.74	0.03	0.02	0.00

MAE: mean absolute error; out of CI: proportion outside credibility interval (95%HPD). Estimates from 100 replicates.

Table S2. Genetic diversity in the simulated data

θ_0	θ_1	τ	P_{GSM}	H_e		N_a		M		V_a		ΔH	
0.4	4	0.01	0.00	0.66	(0.02)	4.88	(0.22)	0.90	(0.03)	1.98	(0.44)	0.13	(0.02)
0.4	40	0.01	0.00	0.88	(0.01)	11.73	(0.47)	0.75	(0.03)	20.65	(4.58)	0.06	(0.01)
0.4	400	0.01	0.00	0.95	(0.00)	22.99	(0.60)	0.49	(0.03)	192.83	(46.27)	0.01	(0.00)
0.4	4	0.05	0.00	0.61	(0.02)	4.26	(0.19)	0.86	(0.04)	1.84	(0.45)	0.13	(0.02)
0.4	40	0.05	0.00	0.84	(0.01)	9.41	(0.34)	0.65	(0.04)	19.26	(4.35)	0.08	(0.01)
0.4	400	0.05	0.00	0.92	(0.00)	16.96	(0.43)	0.38	(0.02)	196.89	(42.34)	0.03	(0.00)
0.4	4	0.1	0.00	0.56	(0.03)	3.80	(0.19)	0.83	(0.04)	1.72	(0.51)	0.13	(0.02)
0.4	40	0.1	0.00	0.79	(0.01)	7.61	(0.33)	0.57	(0.03)	18.32	(4.82)	0.10	(0.01)
0.4	400	0.1	0.00	0.89	(0.01)	12.70	(0.39)	0.31	(0.02)	183.03	(42.65)	0.05	(0.01)
0.4	4	0.5	0.00	0.36	(0.04)	2.59	(0.15)	0.86	(0.04)	0.75	(0.27)	0.08	(0.03)
0.4	40	0.5	0.00	0.52	(0.03)	3.55	(0.20)	0.48	(0.05)	9.18	(3.45)	0.11	(0.03)
0.4	400	0.5	0.00	0.64	(0.02)	4.76	(0.25)	0.19	(0.02)	120.09	(34.76)	0.12	(0.02)
4	0.4	0.01	0.00	0.26	(0.04)	2.27	(0.13)	0.99	(0.02)	0.20	(0.05)	0.02	(0.03)
40	0.4	0.01	0.00	0.26	(0.04)	2.34	(0.15)	0.98	(0.02)	0.21	(0.06)	0.01	(0.03)
400	0.4	0.01	0.00	0.27	(0.04)	2.35	(0.14)	0.98	(0.02)	0.20	(0.05)	0.02	(0.03)
4	0.4	0.05	0.00	0.29	(0.03)	2.61	(0.14)	0.99	(0.01)	0.22	(0.05)	0.00	(0.03)
40	0.4	0.05	0.00	0.31	(0.03)	2.85	(0.14)	0.99	(0.01)	0.24	(0.05)	-0.01	(0.03)
400	0.4	0.05	0.00	0.32	(0.04)	2.97	(0.15)	0.99	(0.01)	0.26	(0.06)	-0.02	(0.03)
4	0.4	0.1	0.00	0.32	(0.03)	2.84	(0.14)	0.99	(0.01)	0.26	(0.06)	0.00	(0.03)
40	0.4	0.1	0.00	0.34	(0.03)	3.17	(0.13)	0.99	(0.01)	0.29	(0.06)	-0.02	(0.03)
400	0.4	0.1	0.00	0.36	(0.03)	3.34	(0.16)	0.99	(0.01)	0.30	(0.06)	-0.02	(0.02)
4	0.4	0.5	0.00	0.47	(0.03)	3.52	(0.16)	0.99	(0.01)	0.46	(0.08)	0.07	(0.03)
40	0.4	0.5	0.00	0.55	(0.02)	4.26	(0.16)	0.99	(0.01)	0.59	(0.07)	0.07	(0.02)
400	0.4	0.5	0.00	0.58	(0.02)	4.54	(0.17)	0.99	(0.01)	0.63	(0.07)	0.07	(0.02)
4			0.00	0.67	(0.02)	5.11	(0.23)	0.92	(0.03)	2.03	(0.47)	0.12	(0.02)
40			0.00	0.89	(0.01)	12.50	(0.46)	0.78	(0.04)	20.28	(4.17)	0.05	(0.01)
400			0.00	0.96	(0.00)	25.94	(0.70)	0.53	(0.03)	202.31	(44.35)	0.01	(0.00)
0.4	4	0.01	0.22	0.69	(0.02)	5.73	(0.28)	0.80	(0.04)	3.96	(0.97)	0.10	(0.02)
0.4	40	0.01	0.22	0.90	(0.01)	14.02	(0.50)	0.65	(0.03)	38.67	(8.66)	0.04	(0.00)
0.4	400	0.01	0.22	0.96	(0.00)	26.23	(0.59)	0.39	(0.02)	394.99	(76.53)	0.01	(0.00)
0.4	4	0.05	0.22	0.65	(0.02)	4.88	(0.24)	0.74	(0.04)	3.75	(0.99)	0.12	(0.02)
0.4	40	0.05	0.22	0.86	(0.01)	10.82	(0.37)	0.54	(0.04)	38.77	(9.79)	0.06	(0.01)
0.4	400	0.05	0.22	0.93	(0.00)	18.48	(0.45)	0.29	(0.02)	394.12	(93.64)	0.02	(0.00)
0.4	4	0.1	0.22	0.60	(0.03)	4.27	(0.25)	0.70	(0.04)	3.54	(0.91)	0.12	(0.02)
0.4	40	0.1	0.22	0.81	(0.01)	8.48	(0.29)	0.46	(0.03)	36.33	(8.69)	0.09	(0.01)
0.4	400	0.1	0.22	0.89	(0.01)	13.57	(0.49)	0.23	(0.02)	369.57	(92.42)	0.04	(0.01)
0.4	4	0.5	0.22	0.39	(0.04)	2.76	(0.19)	0.73	(0.06)	1.58	(0.70)	0.08	(0.03)
0.4	40	0.5	0.22	0.54	(0.03)	3.74	(0.23)	0.37	(0.04)	18.87	(5.98)	0.11	(0.02)
0.4	400	0.5	0.22	0.64	(0.03)	4.87	(0.29)	0.14	(0.02)	237.61	(76.26)	0.11	(0.02)
4	0.4	0.01	0.22	0.27	(0.04)	2.44	(0.16)	0.88	(0.04)	0.41	(0.14)	0.01	(0.03)
40	0.4	0.01	0.22	0.28	(0.04)	2.54	(0.17)	0.87	(0.04)	0.42	(0.13)	0.00	(0.03)
400	0.4	0.01	0.22	0.27	(0.04)	2.56	(0.18)	0.87	(0.04)	0.41	(0.13)	-0.01	(0.03)
4	0.4	0.05	0.22	0.30	(0.04)	2.93	(0.19)	0.88	(0.04)	0.46	(0.13)	-0.03	(0.03)
40	0.4	0.05	0.22	0.31	(0.03)	3.22	(0.19)	0.89	(0.04)	0.48	(0.15)	-0.05	(0.03)
400	0.4	0.05	0.22	0.33	(0.03)	3.46	(0.21)	0.89	(0.03)	0.51	(0.14)	-0.07	(0.03)
4	0.4	0.1	0.22	0.34	(0.04)	3.23	(0.19)	0.88	(0.03)	0.53	(0.14)	-0.03	(0.03)
40	0.4	0.1	0.22	0.36	(0.04)	3.79	(0.19)	0.89	(0.03)	0.57	(0.14)	-0.07	(0.03)
400	0.4	0.1	0.22	0.38	(0.03)	4.13	(0.20)	0.91	(0.03)	0.58	(0.13)	-0.09	(0.03)
4	0.4	0.5	0.22	0.50	(0.03)	4.25	(0.20)	0.89	(0.03)	0.94	(0.20)	0.02	(0.03)
40	0.4	0.5	0.22	0.58	(0.02)	5.50	(0.24)	0.91	(0.02)	1.15	(0.16)	0.01	(0.02)
400	0.4	0.5	0.22	0.61	(0.02)	6.10	(0.22)	0.92	(0.02)	1.26	(0.16)	0.00	(0.02)
4			0.22	0.70	(0.02)	6.13	(0.28)	0.81	(0.04)	4.03	(1.02)	0.09	(0.02)
40			0.22	0.91	(0.01)	15.38	(0.56)	0.69	(0.03)	40.41	(8.88)	0.04	(0.00)
400			0.22	0.97	(0.00)	30.05	(0.79)	0.44	(0.03)	404.74	(87.05)	0.01	(0.00)
0.4	4	0.01	0.74	0.76	(0.02)	8.13	(0.36)	0.35	(0.03)	53.39	(14.15)	0.04	(0.02)
0.4	40	0.01	0.74	0.95	(0.00)	21.55	(0.49)	0.29	(0.02)	500.67	(113.82)	0.01	(0.00)
0.4	400	0.01	0.74	0.98	(0.00)	33.96	(0.56)	0.14	(0.01)	5036.43	(1066.17)	0.00	(0.00)
0.4	4	0.05	0.74	0.71	(0.02)	6.42	(0.30)	0.32	(0.03)	45.34	(11.30)	0.08	(0.02)
0.4	40	0.05	0.74	0.90	(0.01)	14.55	(0.42)	0.21	(0.02)	508.76	(117.72)	0.04	(0.00)
0.4	400	0.05	0.74	0.95	(0.00)	21.98	(0.44)	0.10	(0.01)	4890.30	(1116.76)	0.01	(0.00)
0.4	4	0.1	0.74	0.66	(0.03)	5.37	(0.28)	0.28	(0.02)	43.68	(10.40)	0.09	(0.02)
0.4	40	0.1	0.74	0.85	(0.01)	10.50	(0.34)	0.17	(0.01)	446.46	(114.97)	0.06	(0.01)
0.4	400	0.1	0.74	0.91	(0.00)	15.31	(0.37)	0.08	(0.01)	4573.37	(1042.02)	0.03	(0.00)
0.4	4	0.5	0.74	0.41	(0.04)	3.11	(0.22)	0.30	(0.03)	20.18	(8.18)	0.06	(0.03)
0.4	40	0.5	0.74	0.56	(0.03)	4.07	(0.25)	0.12	(0.02)	232.19	(82.91)	0.10	(0.02)
0.4	400	0.5	0.74	0.66	(0.02)	5.17	(0.27)	0.04	(0.01)	3327.71	(1235.35)	0.10	(0.02)
4	0.4	0.01	0.74	0.29	(0.04)	2.79	(0.24)	0.40	(0.04)	5.01	(2.09)	-0.02	(0.03)
40	0.4	0.01	0.74	0.29	(0.04)	2.87	(0.22)	0.40	(0.04)	5.27	(2.38)	-0.03	(0.03)
400	0.4	0.01	0.74	0.29	(0.04)	2.91	(0.25)	0.40	(0.04)	5.25	(2.27)	-0.04	(0.03)
4	0.4	0.05	0.74	0.32	(0.04)	3.42	(0.27)	0.39	(0.04)	6.26	(2.49)	-0.07	(0.03)
40	0.4	0.05	0.74	0.34	(0.03)	4.07	(0.26)	0.40	(0.03)	6.30	(2.14)	-0.12	(0.03)
400	0.4	0.05	0.74	0.35	(0.04)	4.45	(0.29)	0.41	(0.03)	6.21	(1.94)	-0.15	(0.03)
4	0.4	0.1	0.74	0.35	(0.04)	4.05	(0.27)	0.40	(0.03)	6.55	(2.10)	-0.11	(0.03)
40	0.4	0.1	0.74	0.38	(0.04)	5.09	(0.30)	0.42	(0.03)	7.32	(2.93)	-0.17	(0.03)
400	0.4	0.1	0.74	0.40	(0.04)	6.00	(0.34)	0.43	(0.03)	7.78	(2.62)	-0.20	(0.03)
4	0.4	0.5	0.74	0.55	(0.03)	6.01	(0.34)	0.41	(0.03)	12.38	(3.70)	-0.06	(0.02)
40	0.4	0.5	0.74	0.64	(0.02)	9.59	(0.39)	0.49	(0.03)	14.46	(2.68)	-0.13	(0.02)
400	0.4	0.5	0.74	0.67	(0.02)	11.80	(0.39)	0.52	(0.02)	16.03	(2.55)	-0.15	(0.02)
4			0.74	0.78	(0.02)	9.01	(0.38)	0.38	(0.03)	49.82	(14.84)	0.03	(0.01)
40			0.74	0.96	(0.00)	25.06	(0.60)	0.32	(0.02)	504.85	(107.93)	0.01	(0.00)
400			0.74	0.99	(0.00)	40.89	(0.52)	0.17	(0.01)	5229.17	(988.28)	0.00	(0.00)

H_e, expected heterozygosity; N_a, number of alleles; M, Garza and Williamson (2001) statistic; V_a, variance of allele size; ΔH, *Bottleneck* statistic (Cornuet and Luikart, 1996). Estimates of genetic diversity are mean values from the 100 simulated data sets for each combination of demographic parameters (θ_0, current scaled effective population size; θ_1, ancestral scaled population size; τ, time since population size change). Standard deviations are shown in parentheses.

Table S3. Parameter estimates from MIGRAINE analysis for real data sets

		ML estimate	95% confidence interval
**whale shark**	$\hat{P}_{GSM}$	0.55	(0.48–0.62)
	$\hat{\theta}_0$	7.47	(3.95–17.85)
	$\hat{\theta}_A$	2.47	(0.01–3.4)
	$\hat{D}$	0.01	(0.001–1.52)
expansion	$\hat{\theta}_{ratio}$	2.47	(1.69–9.01)
**leatherback**	$\hat{P}_{GSM}$	0.62	(0.54–0.69)
**turtle**	$\hat{\theta}_0$	4.90	(3.79–151.8)
	$\hat{\theta}_A$	0.02	($< 5 \times 10^{-3}$–3.42)
	$\hat{D}$	1.07	(1.37×10^{-5}–33.77)
expansion	$\hat{\theta}_{ratio}$	197.7	(1.08–983.7)
**black-and-white**	$\hat{P}_{GSM}$	0.31	(0.15–0.46)
**colobus**	$\hat{\theta}$	1.40	(0.93–2.09)
constant size	$\hat{\theta}_{ratio}$	113.4	(2.7×10^{-5}–585)
**red**	$\hat{P}_{GSM}$	0.25	(0–0.49)
**colobus**	$\hat{\theta}_0$	0.83	($< 1 \times 10^{-4}$–2.46)
	$\hat{\theta}_A$	6.10	(2.18–22.41)
	$\hat{D}$	0.26	(0.01–2.25)
contraction	$\hat{\theta}_{ratio}$	0.14	(4.57×10^{-6}–0.73)

Table S4. Genetic diversity in the real data

Species	n	# loci	H_e	N_a	M	V_a	ΔH
whale shark	478	14	0.66	11.57	0.76	7.51	0.02
leatherback turtle	215	10	0.70	12.00	0.80	9.87	0.01
black-and-white colobus	22	14	0.53	3.86	0.76	1.35	0.08
red colobus	23	13	0.68	5.08	0.65	6.04	0.12

n, sample size; H_e, expected heterozygosity; N_a, number of alleles; M, Garza and Williamson (2001) statistic; V_a, variance of allele size; ΔH, *Bottleneck* statistic (Cornuet and Luikart, 1996).

Table S5. Demographic parameter estimates from MIGRAINE analysis for PODs

		ML estimate	95% confidence interval	true value
**contraction**	$\hat{\theta}_0$	0.13	$(1.93 \times 10^{-4}$–$2.16)$	0.4
	$\hat{\theta}_A$	11.28	(4.69–189.1)	40
	$\hat{D}$	0.20	(0.06–0.89)	0.25
	$\hat{\theta}_{ratio}$	0.01	$(7.59 \times 10^{-6}$–$0.26)$	0.01
**expansion**	$\hat{\theta}_0$	36.11	(3.35–445.9)	40
	$\hat{\theta}_A$	0.34	$(2.85 \times 10^{-8}$–$0.79)$	0.4
	$\hat{D}$	0.10	(0.03–1.90)	0.25
	$\hat{\theta}_{ratio}$	105.1	$(9.63$–$4.5 \times 10^{8})$	100
**constant size**	$\hat{\theta}$	47.55	(30.08–88.11)	40
	$\hat{\theta}_{ratio}$	10^8	$(10^{-6}$–$10^{10})$	1

Table S6. Estimation of P_{GSM} with DIYABCskylineplot, MIGRAINE and BEAST for PODs

$P_{GSM} = 0.22$		$\hat{P}_{GSM}$	95% interval
contraction	DIYABCskylineplot	0.60	(0.06–0.80)
	MIGRAINE	0.60	(0.00–0.74)
	BEAST	n.a.	n.a.
expansion	DIYABCskylineplot	0.20	(0.05–0.37)
	MIGRAINE	0.28	(0.14–0.43)
	BEAST	0.29	(0.15–0.43)
constant size	DIYABCskylineplot	0.18	(0.01–0.45)
	MIGRAINE	0.21	(0.00–0.37)
	BEAST	0.55	(0.45–0.64)

Table S7. Bayes factors of constant *versus* variable effective population size for PODs

		Bayes factor	evidence against constant size
contraction	DIYABCskylineplot	8.71	substantial
	BEAST	> 1000	decisive
expansion	DIYABCskylineplot	249	decisive
	BEAST	999	decisive
constant size	DIYABCskylineplot	0.77	negative
	BEAST	124	decisive

Figure S2. ABC Skyline Plots: contractions, $P_{GSM} = 0.00$. Superimposed skyline plots (median in black, and 95%HPD interval in grey of the posterior probability distribution for $\theta(t)$) from 100 replicates for each scenario of demographic contraction and mutational model with $P_{GSM} = 0.00$. True demography is shown in orange.

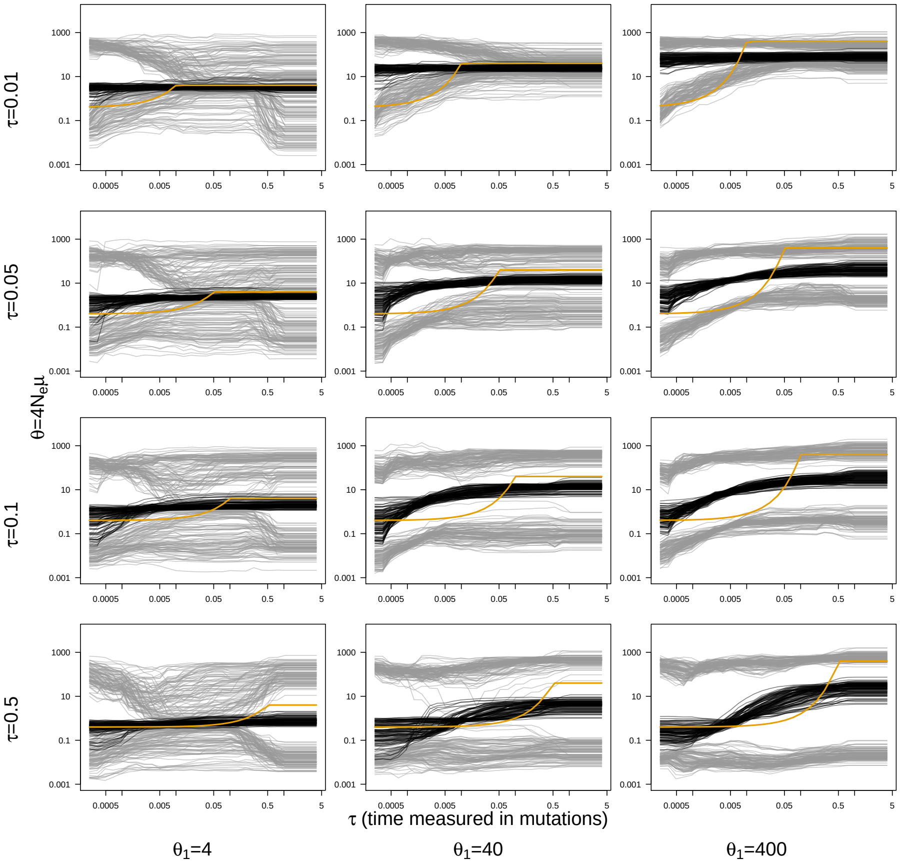

Figure S3. ABC Skyline Plots: contractions, $P_{GSM} = 0.22$. Superimposed skyline plots (median in black, and 95%HPD interval in grey of the posterior probability distribution for $\theta(t)$) from 100 replicates for each scenario of demographic contraction and mutational model with $P_{GSM} = 0.22$. True demography is shown in orange.

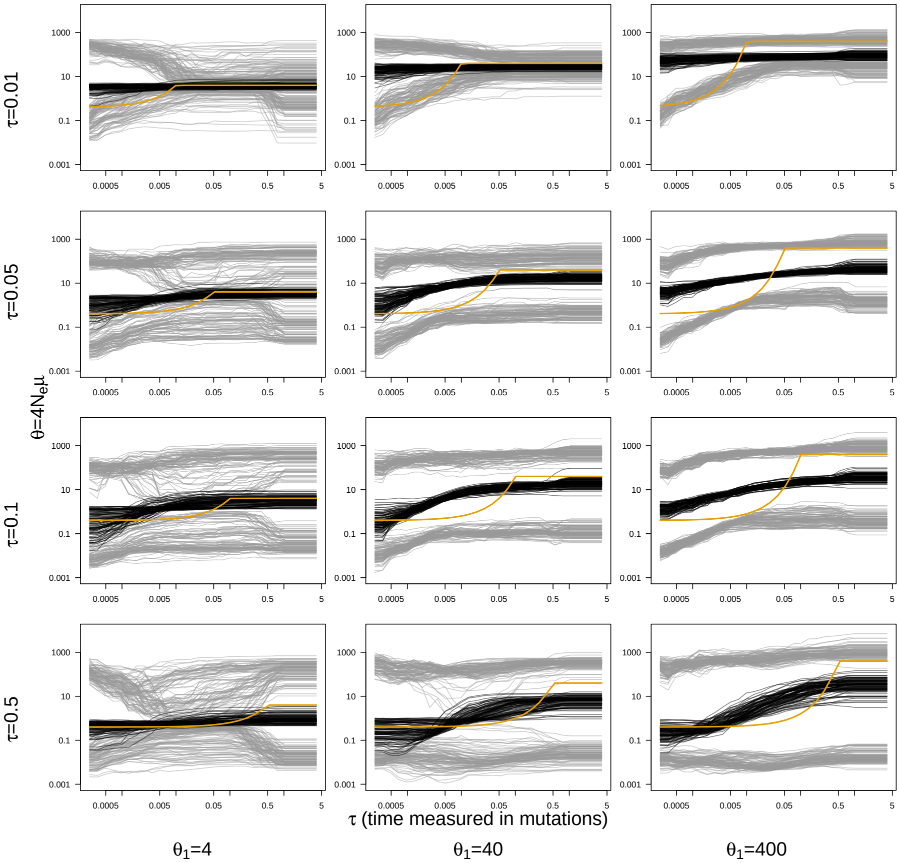

Figure S4. ABC Skyline Plots: contractions, $P_{GSM} = 0.74$.
Superimposed skyline plots (median in black, and 95%HPD interval in grey of the posterior probability distribution for $\theta(t)$) from 100 replicates for each scenario of demographic contraction and mutational model with $P_{GSM} = 0.74$. True demography is shown in orange.

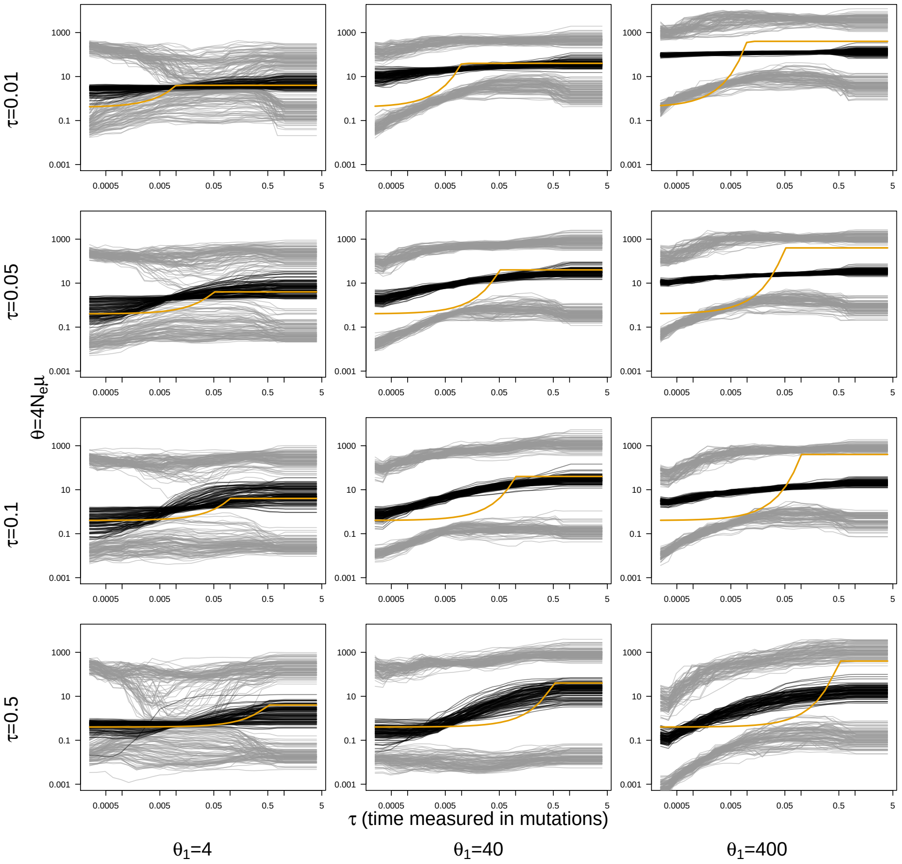

Figure S5. ABC Skyline Plots: expansions, $P_{GSM} = 0.00$.
Superimposed skyline plots (median in black, and 95%HPD interval in grey of the posterior probability distribution for $\theta(t)$) from 100 replicates for each scenario of demographic expansion and mutational model with $P_{GSM} = 0.00$. True demography is shown in orange.

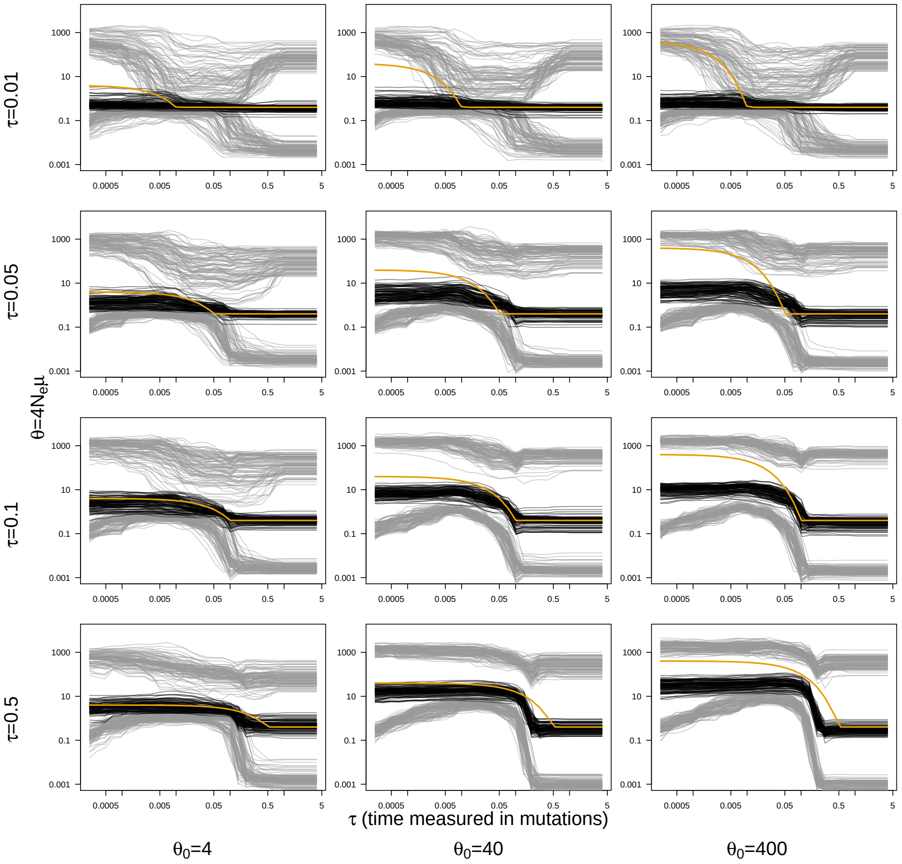

Figure S6. ABC Skyline Plots: expansions, $P_{GSM} = 0.22$.
Superimposed skyline plots (median in black, and 95%HPD interval in grey of the posterior probability distribution for $\theta(t)$) from 100 replicates for each scenario of demographic expansion and mutational model with $P_{GSM} = 0.22$. True demography is shown in orange.

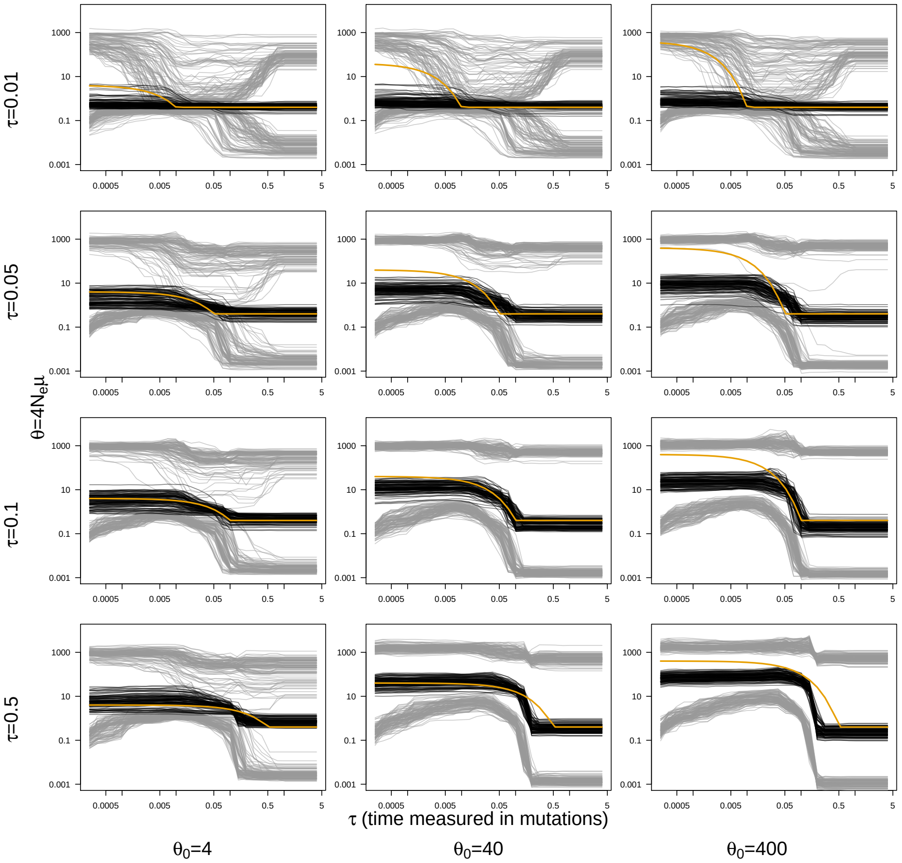

Figure S7. ABC Skyline Plots: expansions, $P_{GSM} = 0.74$.
Superimposed skyline plots (median in black, and 95%HPD interval in grey of the posterior probability distribution for $\theta(t)$) from 100 replicates for each scenario of demographic expansion and mutational model with $P_{GSM} = 0.74$. True demography is shown in orange.

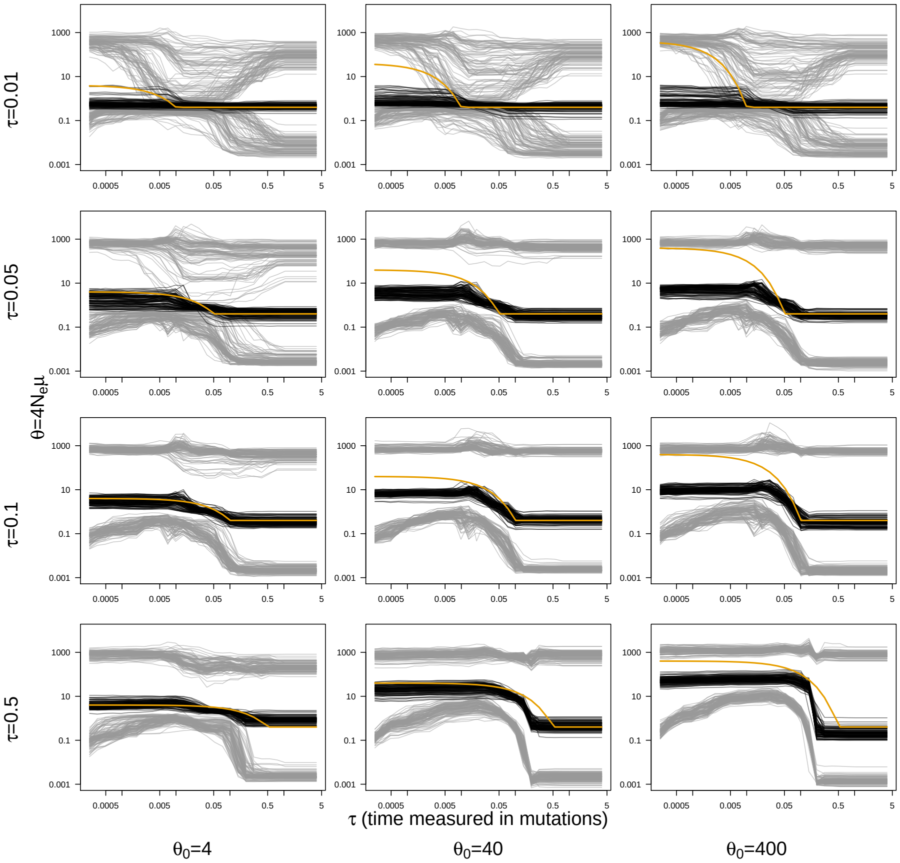

Figure S8. ABC Skyline Plots: constant size. Superimposed skyline plots (median in black, and 95%HPD interval in grey of the posterior probability distribution for $\theta(t)$) from 100 replicates for each scenario of constant size. True demography is shown in orange.

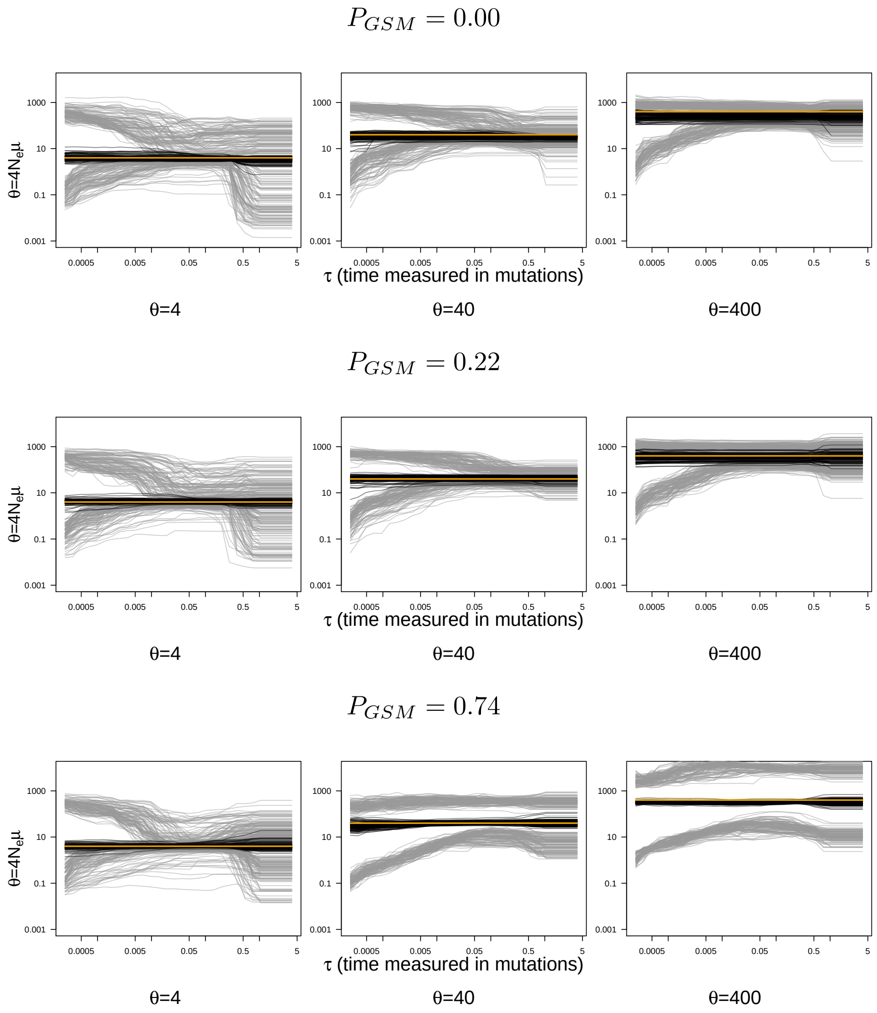

Figure S9. Evidence for variable population size: contractions, $P_{GSM} = 0.00$. Bayes factor distribution (boxplot) from 100 replicates for each scenario of demographic contraction and mutational model with $P_{GSM} = 0.00$. For reference, Jeffreys scale is given for the evidence against constant size.

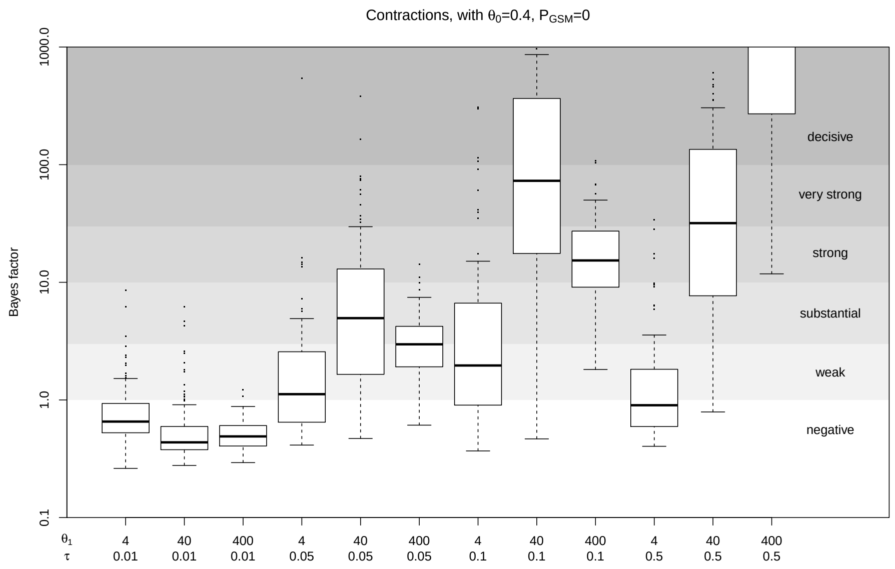

Figure S10. Evidence for variable population size: contractions, $P_{GSM} = 0.22$. Bayes factor distribution (boxplot) from 100 replicates for each scenario of demographic contraction and mutational model with $P_{GSM} = 0.22$. For reference, Jeffreys scale is given for the evidence against constant size.

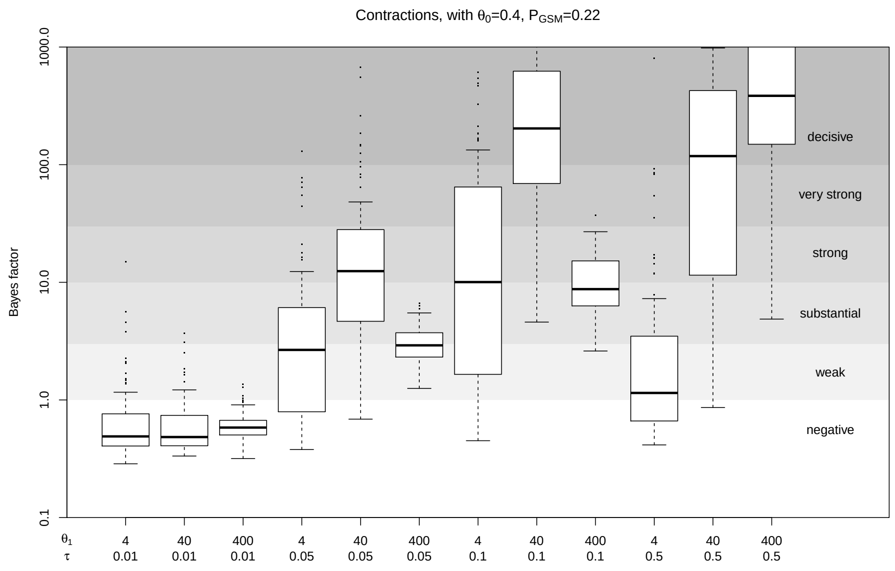

Figure S11. Evidence for variable population size: contractions, $P_{GSM} = 0.74$. Bayes factor distribution (boxplot) from 100 replicates for each scenario of demographic contraction and mutational model with $P_{GSM} = 0.74$. For reference, Jeffreys scale is given for the evidence against constant size.

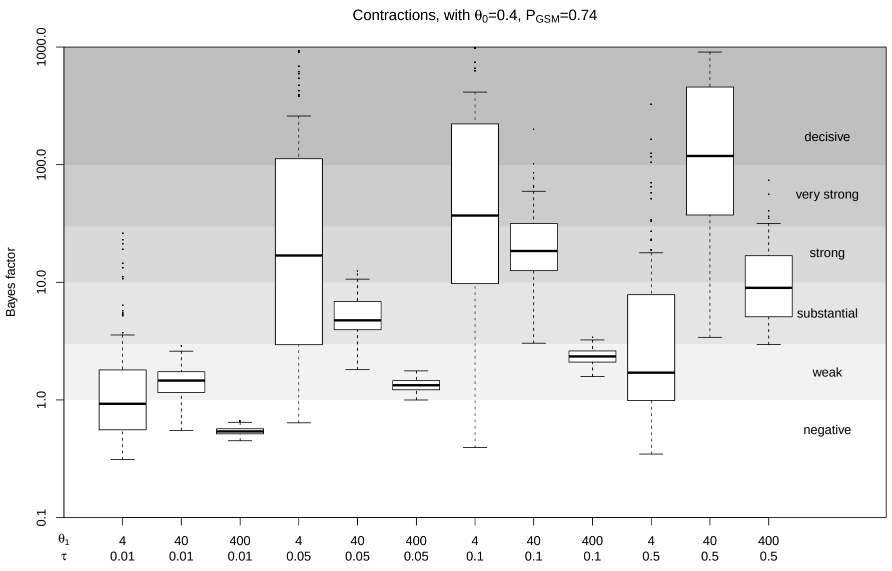

Figure S12. Evidence for variable population size: expansions, $P_{GSM} = 0.00$. Bayes factor distribution (boxplot) from 100 replicates for each scenario of demographic expansion and mutational model with $P_{GSM} = 0.00$. For reference, Jeffreys scale is given for the evidence against constant size.

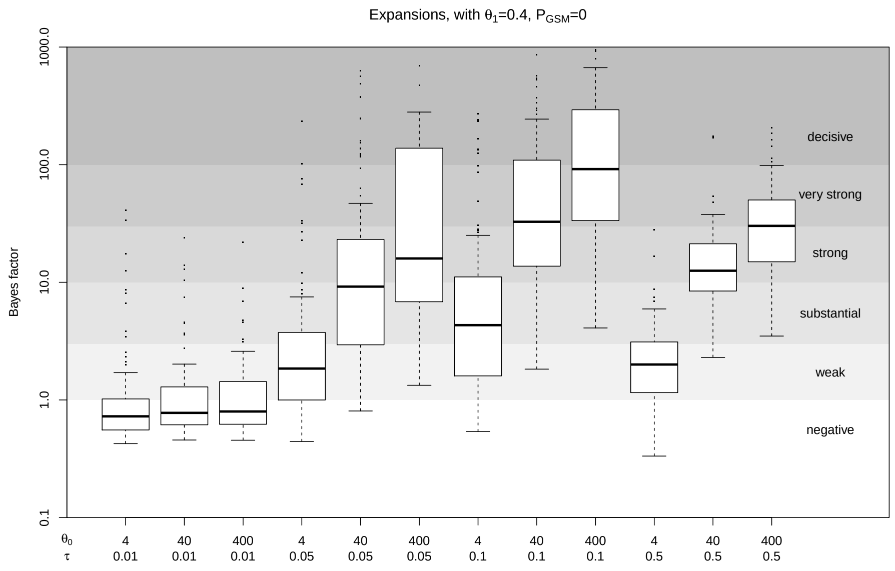

Figure S13. Evidence for variable population size: expansions, $P_{GSM} = 0.22$. Bayes factor distribution (boxplot) from 100 replicates for each scenario of demographic expansion and mutational model with $P_{GSM} = 0.22$. For reference, Jeffreys scale is given for the evidence against constant size. Arrows indicate scenarios for which replicates had BF superior to 1000.

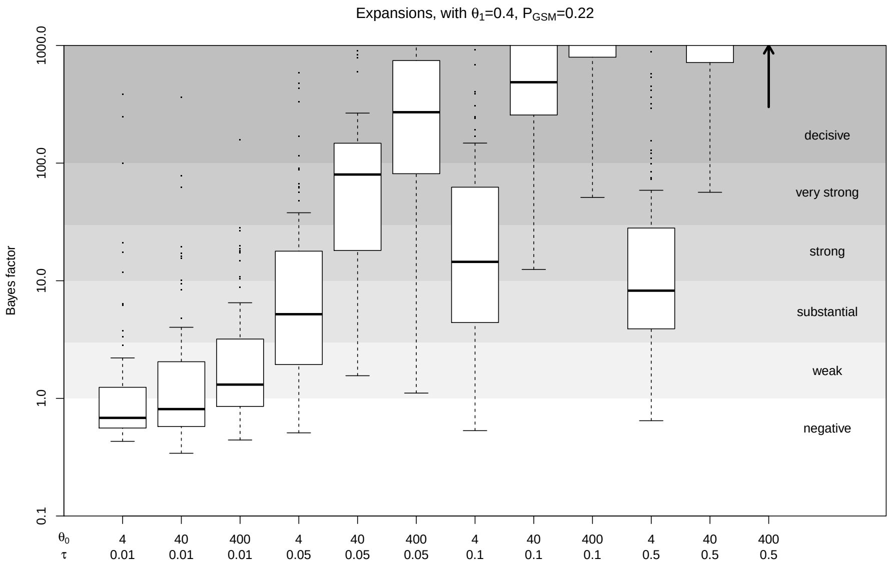

Figure S14. Evidence for variable population size: expansions, $P_{GSM} = 0.74$. Bayes factor distribution (boxplot) from 100 replicates for each scenario of demographic expansion and mutational model with $P_{GSM} = 0.74$. For reference, Jeffreys scale is given for the evidence against constant size. Arrows indicate scenarios for which replicates had BF superior to 1000.

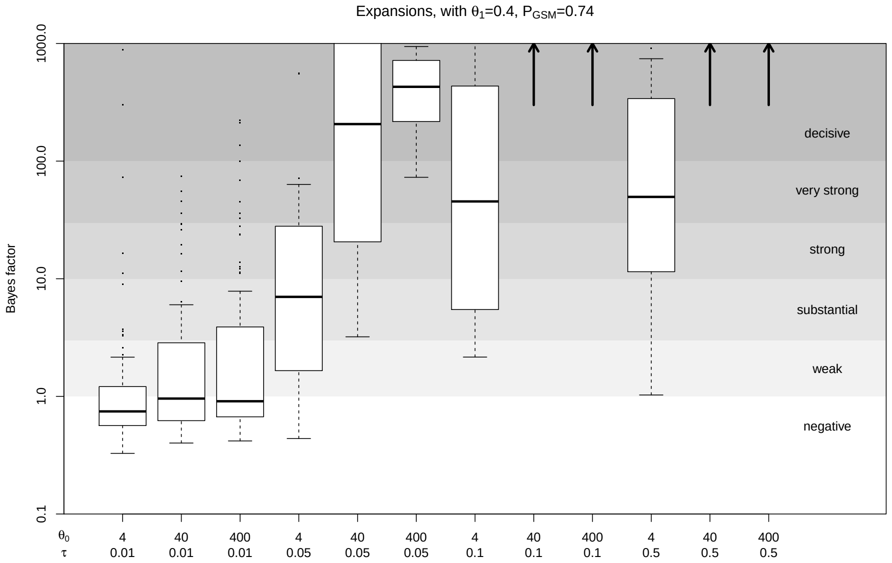

Figure S15. Evidence for variable population size: constant population size. Bayes factor distribution (boxplot) from 100 replicates for each scenario of constant size. For reference, Jeffreys scale is given for the evidence against constant size.

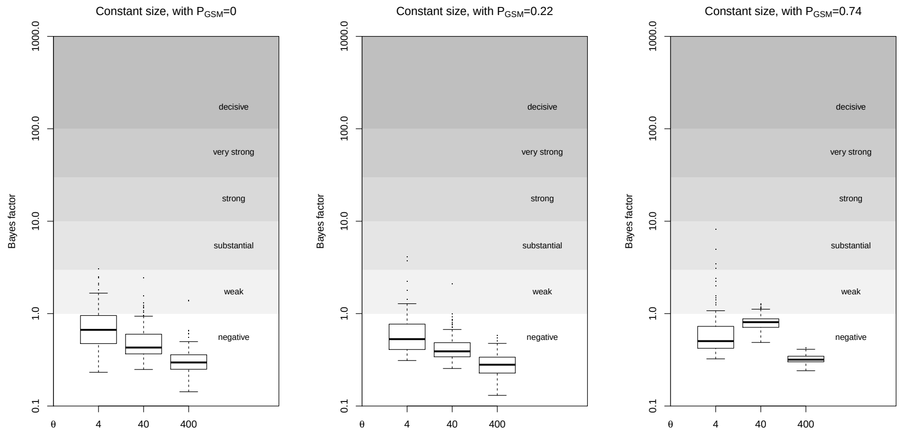

Figure S16. Prior and posterior probability distributions for parameter P_{GSM}. Parameter values sampled from the prior distribution are plotted in the grey histogram and regression adjusted values of the parameter from the retained simulations are represented in the red histogram.

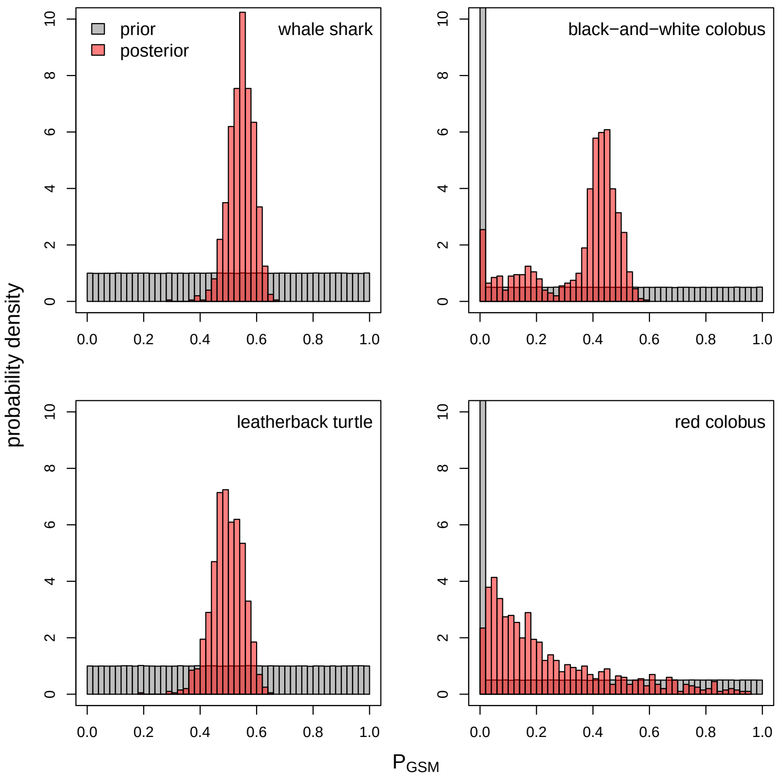

Figure S17. Alternative ABC Skyline plots. Superimposed skyline plots (median in black, and 95%HPD interval in grey of the posterior probability distribution for $\theta(t)$) from 100 replicates for example contraction ($\theta_0 = 0.4$, $\theta_1 = 40$, $\tau = 0.1$), expansion ($\theta_0 = 40$, $\theta_1 = 0.4$, $\tau = 0.1$) and constant size ($\theta = 40$) scenarios with mutational model $P_{GSM} = 0.22$. True demography is shown in orange. Note that present is at $\tau = 0$ (left). These estimates are obtained through the alternative ABC skyline plot approach described in the Supplementary Methods S1.2.

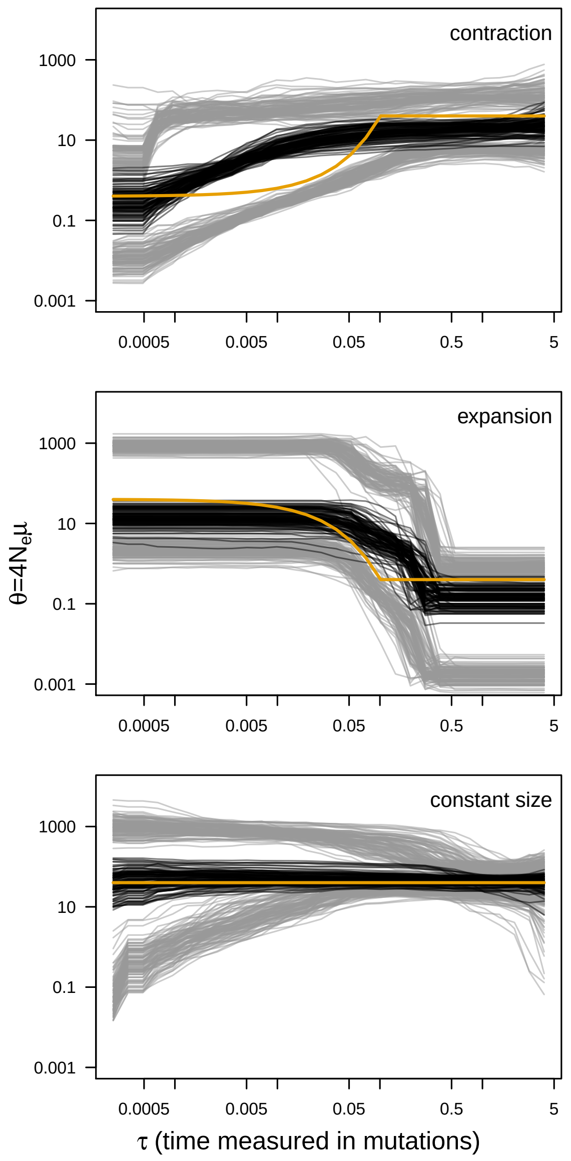

Figure S18. Detection of demographic change with the ratio θ_0/θ_1 in ABC. Point estimate (median, represented with black dots) and 95% credibility intervals (grey bars) for the ratio θ_0/θ_1, from 100 replicates for example contraction ($\theta_0 = 0.4$, $\theta_1 = 40$, $\tau = 0.1$), expansion ($\theta_0 = 40$, $\theta_1 = 0.4$, $\tau = 0.1$) and constant size ($\theta = 40$) scenarios with mutational model $P_{GSM} = 0.22$. Position of replicates in the abscissa is arbitrary. Credibility intervals not containing the value $\theta_0 = \theta_1$ (black horizontal line) will be considered as evidence for expansion or contraction. These estimates are obtained through the alternative ABC skyline plot approach described in the Supplementary Methods S1.2.

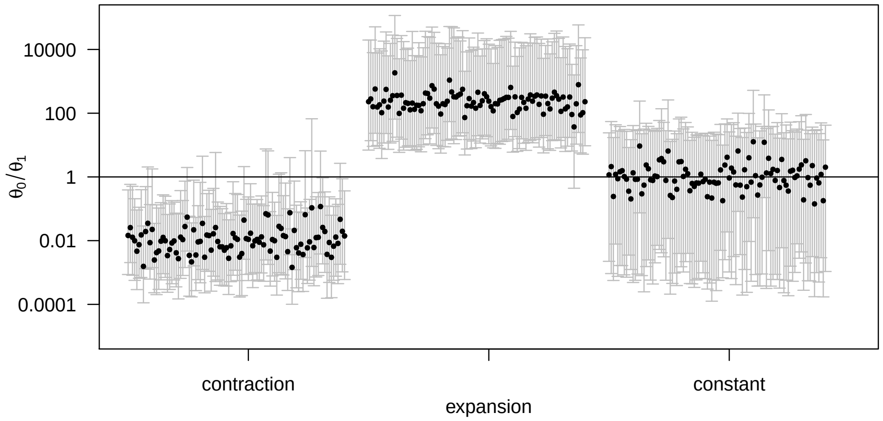

Figure S19. Comparison of methods on three simulated datasets. Black lines: skyline plots (median and 95%HPD interval of the posterior probability distribution) obtained with DIYABCskylineplot. Blue lines: skyline plots (median and 90%HPD interval of the posterior probability distribution) obtained with VarEff. Pink lines: skyline plots (median and 95%HPD interval of the posterior probability distribution) obtained with BEAST. Demographic trajectories based on parameters point estimates from MIGRAINE analysis are shown with a green line for reference. True demography is shown in orange. Note that present is at $\tau = 0$ (left).

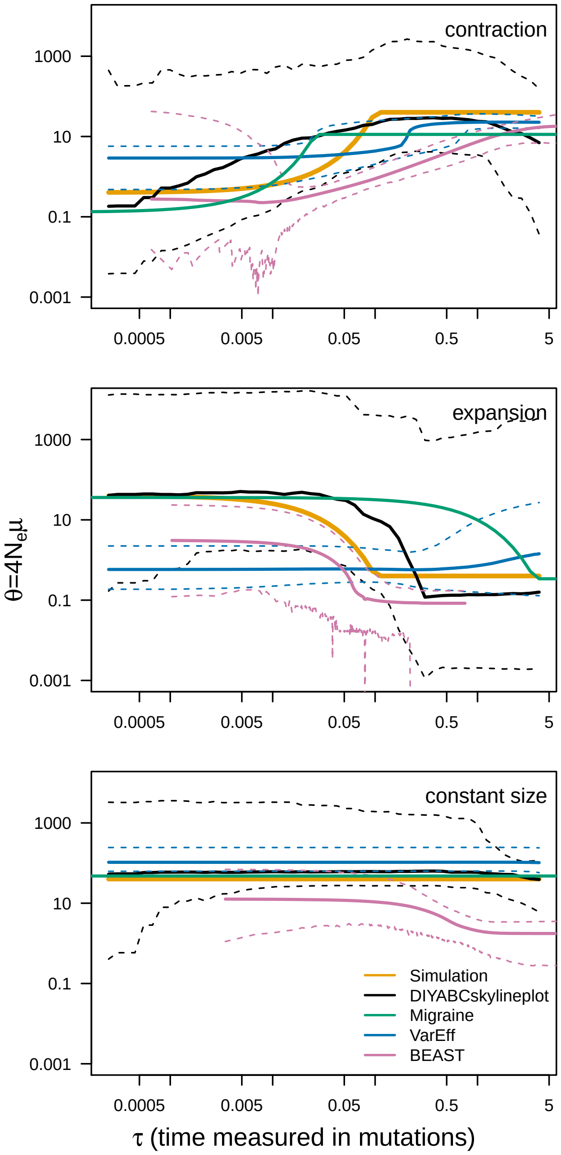

Figure S20. Comparison of methods on real data. Demographic inference for the whale shark, leatherback turtle, Western black-and-white colobus and Temminck's red colobus. Black lines: skyline plots (median and 95%HPD interval of the posterior probability distribution) obtained with DIYABCskylineplot. Blue lines: skyline plots (median and 90%HPD interval of the posterior probability distribution) obtained with VarEff. Pink lines: skyline plots (median and 95%HPD interval of the posterior probability distribution) obtained with BEAST. Demographic trajectories based on parameters point estimates from MIGRAINE analysis are shown with a green line for reference. Note that present is at $\tau = 0$ (left).

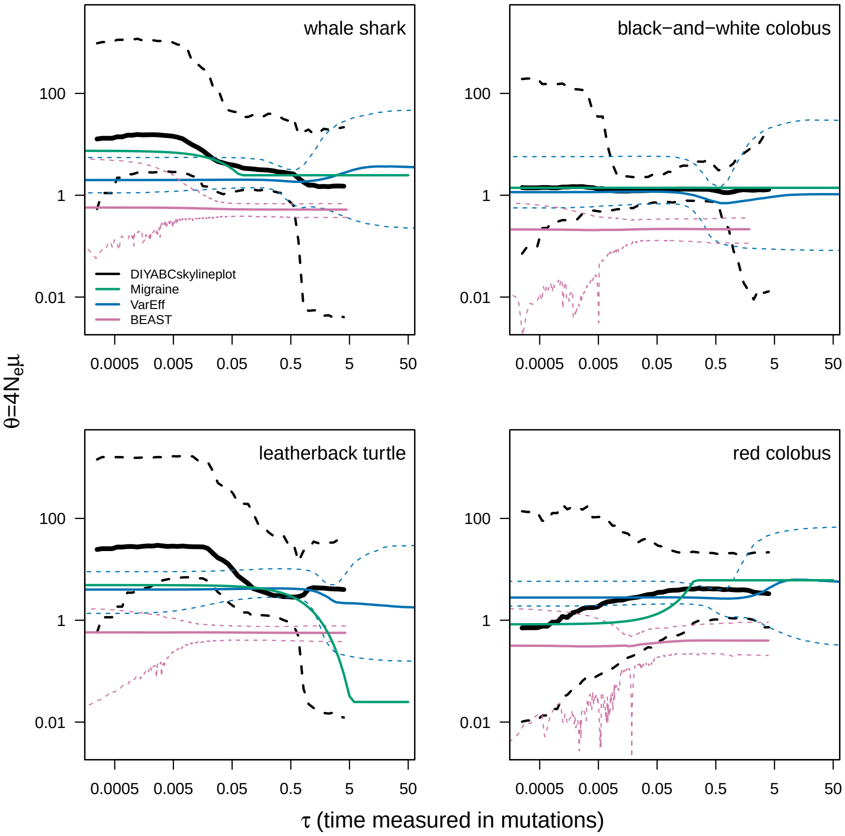

Supplement: Supplemental Information 1 [file peerj-05-3530-s001.pdf]
